# Designing mindfulness information for interaction in social media: The role of information framing, health risk perception and lay theories of health

**DOI:** 10.3389/fpsyg.2022.1041016

**Published:** 2023-01-04

**Authors:** Xiaomei Wang, Bin Zhu, Qing Guo, Wenyu Wang, Ruoxuan Zhao

**Affiliations:** ^1^School of Media Studies and Humanities, Zhejiang University City College, Hangzhou, Zhejiang, China; ^2^College of Media and International Culture, Zhejiang University, Hangzhou, Zhejiang, China

**Keywords:** mindfulness information, information framing, health risk perception, lay theories of health, health information

## Abstract

In the post-pandemic era, our health is facing unprecedented challenges, and people are more willing to obtain health-related information or interact with each other than ever before. In this context, people’s interest in mindfulness information is also growing. However, not enough attention has been paid to the relationship between mindfulness information design and information interaction. The purpose of this study is to assess the impact of information design based on the gain and loss framework on people’s willingness to interact with mindfulness information, and to identify the framework for achieving better results. Through two experimental studies, we find that information design based on the framework of gains and losses can produce different effects. Specifically, the findings of the first experiment (*N* = 282) shows the individuals are more willing to interact mindfulness information when they are exposed to gain-framed information rather than loss-framed. In the second experiment (*N* = 308), we find that loss framing, compared with gain framing, led to greater health risk perception, which in turn make participants more likely to interact with mindfulness information with others. Additionally, our results show that the lay theories of health plays a moderating role in the direct effect of information framework on willingness to interact with mindfulness information in social media. When individuals hold incremental lay theories, they are more willing to interact with mindfulness information under the gain-framed information condition compared with the loss-framed information condition. However, when individuals are in entity condition, there is no significant difference in the willingness to interact with mindfulness information between the gain-framed and loss-framed information. Our studies of integrating information framework into designing mindfulness information suggest a promising strategy of health information interaction in social media.

## 1. Introduction

In the post-pandemic era, we are forced to embrace uncertainty and challenges. There is increasing interest in mindfulness for its potential benefits as a wellbeing practice to improve quality of life (Mars and Abbey, 2010; de Frias and Whyne, 2015; Behan, 2020). The mindfulness movement, which advocates viewing the body, self and life with an accepting and non-judgmental attitude, brings a perspective of attentive awareness on the present moment. While encouraging people to face the uncertainty and impermanence in life, mindfulness brings them to a centered calm without being carried away by negative emotions (Kabat-Zinn and Hanh, 2009). In other words, mindfulness allows people to accept life situation and bodily state with less criticism and judgment.

Over the last few years, the different aspects and dimensions of mindfulness have been touched upon integrating with various disciplines, such as neuroscience, psychology, physiology, art, design and media, to create novel communication and interaction (Vidyarthi et al., 2012; Rheden and Hengeveld, 2016). With the development of media technology, people are more and more accustomed to obtaining health-related information and knowledge from new media. Media technologies related to mindfulness and relevant research endeavors have increased markedly, as can be seen from the growing number of academic publications (Terzimehić et al., 2019; Lukoff et al., 2020; Liu et al., 2022a). These studies provided with diverse perspectives and foundations for investigating health information about mindfulness.

With the widespread popularity of mobile networks, people are more likely to use mobile phones to obtain health information (Liu et al., 2022b). Social media platforms have become an important medium for people to search, share, and discuss information (Choi and Toma, 2014; Muir et al., 2020). In this context, many scholars have begun to explore how to achieve health interventions for target groups through the dissemination and guidance of health information. However, in the field of health communication research, many studies are concerned with the influence of health information content on individual health behaviors (Schubbe et al., 2020). However, the relationship between health information design and health behavior has not been paid enough attention. Researchers have found subtle changes in the presentation of information frames cause individuals to choose different action responses (Rothman and Salovey, 1997; Nan, 2012). In other words, the effectiveness of persuasion in promoting behavior change may depend on the way the information is designed, not just the meaning of the content itself. From this sense, health information design takes an important role. For instance, frame is a kind of information customization method, which can influence individual’s behavior decision by adjusting the expression of information without changing the meaning of content, so as to promote specific behavior (Rothman and Salovey, 1997). It is found that people’s decision-making and behavior preferences are often influenced by the way of information expression (Akl et al., 2011). Berger and Milkman (2012) have suggested that positive information is more communicative than negative information. They found that most people prefer to be seen as someone who shares optimistic stories or makes others feel good, rather than sharing things that make others sad or upset. Moreover, sharing positive information can also help boost the mood of others or provide information about potential returns. When people are exposed to the negative consequences of behavior, they tend to seek risks, while those exposed to the positive consequences are more risk-averse (Kahneman and Tversky, 2013). It is hard to achieve the ideal intervention effect if only paying attention to the information content. Therefore, grasping the interaction process between information and people by designing a reasonable information framework to influence their behavior preferences can achieve a better communication effect (Xu et al., 2021).

However, it is worth noting that many studies are examined the impact of information framework on health-related behaviors (Rothman and Salovey, 1997; Kim and Lee, 2017; Gao et al., 2022), rather than the willingness to interact with mindfulness information. On the other hand, some key methodological challenges and issues need to be solved in this area. One of the crucial issues is the design of mindfulness information for dissemination through mobile social media based on individual differences (Davidson and Dahl, 2018). Therefore, the purpose of this study was to gain a better understanding of the information characteristics that contribute to effective strategies of designing mindfulness information for a better result of dissemination. By using the lens of framing effect theory and lay theory, we explored the relationship between mindfulness information framework and willingness to interact with the mindfulness information in social media with a focus on the internal possible mechanism through online control experiments.

## 2. Theoretical background and hypotheses

### 2.1. Framing effect, health behavior and information interactions

Persuasive information is used as a way to motivate individuals’ health behaviors (Xu et al., 2020). One strategy to optimize the effectiveness of information is to use information framework (Lithopoulos et al., 2017). Information framework originated from prospect theory, and its essence is to design the expression of information to change people’s decision preference (Tversky and Kahneman, 1981). Reviewing the past literature, information framework is widely used in health behavior research such as chronic diseases (Gao et al., 2022), HPV vaccination (Xu et al., 2021), smoking (Kim and Lee, 2017). Most health-related information can be explained by benefits or costs. Information framing can play an important role in promoting health behaviors (Akl et al., 2011). Gain-loss framework emphasizes the benefits of engaging in a behavior (gain framework) and the losses caused by not engaging in a behavior (loss framework; Rothman and Salovey, 1997). For example, gain-framed mindfulness information may emphasize the positive outcomes (such as happiness) associated with practicing mindfulness. Meanwhile, a loss-framed mindfulness messages might be framed in relation to negative outcomes(such as anxiety) of without practicing mindfulness. However, as to which information framework is more effective, the research shows inconsistent results. Rothman and Salovey (1997) put forward dual behavior framework of “prevention-detection,” and found that the loss-framed information was more effective in disease detection behavior, while the gain-framed information was more effective in disease prevention behavior. The effectiveness of the message framework depended on the matching between the information and the audience or situation (Rothman et al., 2020). A meta-analytic review found that the gain framework was more convincing than the loss framework in advocating dental hygiene behaviors. However, in terms of other preventive measures (such as skin cancer prevention, or diet and nutrition), there is no statistically significant differences in the persuasiveness between different frame information (O'Keefe and Jensen, 2007). Gallagher and Updegraff (2012) through meta-analysis also found that the benefit frame information is more convincing than the loss frame information in preventing skin cancer, encouraging smoking cessation and physical activities, while in cancer detection and HPV vaccination, the loss framework is more convincing than the benefit framework (Lee-Won et al., 2017; Xu et al., 2020). Some studies also examined the effects of frame information on the promotion of health sports, which showed that the gain-framed information was more likely to inspire people to participate in sports than the loss-framed information (Gallagher and Updegraff, 2012). According to the dual behavior framework of “prevention-detection” (Rothman and Salovey, 1997), mindfulness can be classified as preventive health behavior. Therefore, we can speculate that the gain-framed information was more likely to inspire people to participate in mindfulness exercises than the loss-framed information. According to the Information-Motivation-Behavior-Skills Model(IMB) proposed by Fisher et al., the factors that affect health behavior change can be divided into three parts, namely, information, motivation and behavior skills. Among them, information is the initial condition for taking health behavior, specifically referring to the information related to health promotion (Misovich et al., 2010). Hence, we advocate that people are more inclined to identify with mindfulness-related information under the gain-framed information rather than the loss-framed one. Some early research has verified that the individuals under the positive frame information are more likely to be at the level of advanced needs, while those under the negative frame information are more likely to be at the level of basic needs (Maslow, 1954). When exposed to the gain-framed (positive) mindfulness information, people may be motivated by higher-level social needs and are willing to engage in more social interactions, such as likes, sharing and commenting on information. However, when exposed to the loss-framed (negative) mindfulness information, people may be inspired by lower-level security needs due to perceived risks, and their social communication needs are not high enough. Based on the above, we put forward the following hypothesis:

*H1*: Compared with the loss framework, mindfulness information in the gain framework is more effective to enhance individuals' willingness of interaction about mindfulness information (such as likes, sharing and commenting on information).

### 2.2. The intermediary role of health risk perception

To further illuminate the underlying mechanism of the relationship between information framing and willingness to interact with the mindfulness information, we also investigated the mediating effect of health risk perception. Risk perception is a concept that describes individuals’ cognitive and psychological response to situations in which something of value is threatened (Setbon et al., 2005). Health risk perception consists of two dimensions: perception of susceptibility and perception of severity. More specifically, perceived susceptibility refers to the audience’s subjective perception of their likelihood of falling into a risky situation; while perceived severity accounts for people’s beliefs about the risk of experiencing the threat (Witte, 1994). The two complement each other and work together to explain the audience’s perception of health risks.

When the level of perceived risk is high, people are very sensitive to all kinds of risk information and have a high demand for information, which leads to active communication behavior about the risks. People’s perceived susceptibility to health status after reading information can positively predict the structural viral transmission of information (Meng et al., 2018). As a result, people tend to share risk information with others, and then take various evasive measures to avoid risk harm. In short, risk perception plays an important intermediary role in the public’s risk communication behavior (Zhang et al., 2020). Research on the information effects has shown that there is a positive correlation between perceived risk and willingness to share information with others. People with a higher level of perceived risk are more likely to engage in health protection behaviors (Zhang C. et al., 2021; Zhang N. et al., 2021), and there is a significant correlation between risk perception and self-reported preventive health behaviors (Dryhurst et al., 2020). In addition, researchers also found that the severity of perceived risk was associated with recommended behavioral changes (Rubin et al., 2009). Witte (1994) pointed out that perceived severity was the key factor affecting people’s behavior change, and the perception of risk was a necessary condition for people to take follow-up action. Perceptual susceptibility also often caused people to ease their emotions by sharing information and affected their subsequent intentions and actions (Ho et al., 2008).

Previous studies have found that information framework affects risk perception (Cho and Boster, 2008). Specifically, loss frame might be more persuasive than gain frame and increase the levels of perceived severity (Bosone and Martinez, 2017), in turn, which was associated with the possibility that individuals would share information with others (Kirkpatrick et al., 2021). Risk perception plays an intermediary role (Li and Zhang, 2021).Thus, we put forward the following hypothesis on the basis of literature:

*H2*: Information framework has an indirect impact on the interaction about mindfulness information through risk perception.

### 2.3. The moderating effect of lay theories of health

One important variable to predict people’s engagement in health-related behaviors is lay theories of health (Bunda and Busseri, 2019). The lay theories of health could be divided into incremental and entity theory: individuals with incremental theory tend to think that their health levels can be improved through their own efforts, whereas individuals with entity theory think that their health levels are more influenced by congenital factors and is hard to change (Zhang and Kou, 2022). Previous research demonstrated that those tended to consider health is malleable(incremental)were more likely to engage in health behaviors than those tended to consider health fixed (entity; Bunda and Busseri, 2019). Recent studies have also shown that lay theories of health can predict the possibility of people participating in health protection behaviors mediated by variables that people consider future consequences (Zhang C. et al., 2021; Zhang N. et al., 2021).

As proposed by Bunda and Busseri (2019), individuals’ subjective perceptions of their health status provide valuable information. Belief is the guide of individual actions, which would determine people’s actions. In fact, behavioral intention is best predicted by the strong belief that one can change one’s health through one’s own efforts(conveyed by an incremental lay theory of health) and the perception of self-improvement in the past (Bunda and Busseri, 2019). It is not difficult to infer that individuals who hold incremental lay theories of health have higher self-efficacy and tend to actively regulate health-related behaviors. Indeed, self-efficacy reflects an individual’s sense of control over life. Previous researchers have found that higher self-efficacy are related to greater participation in health-promoting behaviors, and higher exercise intensity and frequency (Luszczynska et al., 2011; Dong et al., 2018). Furthermore, in an experimental study, self-efficacy was positively correlated with incremental health theory which was positively correlated with participation in health-related behavior, but negatively correlated with entity health theory which was negatively correlated with participation in health-related behavior (Zhang C. et al., 2021; Zhang N. et al., 2021). Van’t Riet et al. (2010) examined the influence of self-efficacy on skin self-examination on the effects of different framed skin-cancer detection information. For participants with high self-efficacy, they are more willing to perform skin self-examination when exposed to loss-framed information rather than gain-framed information. For participants with low self-efficacy, there was no difference in intention under the condition of gain-and loss-framed information. According to the aforementioned dual behavioral framework of “prevention - detection” (Rothman and Salovey, 1997), the loss framework was more convincing than the gain framework in disease detection behavior (such as skin cancer detection; Lee-Won et al., 2017; Xu et al., 2020), and individual self-efficacy level moderated the effect of information frame on health-promoting behavior (Van’t Riet et al., 2010). From this, we could speculate that the gain framework might be more convincing than the loss framework in health prevention behavior (such as mindfulness-related behaviors), and the lay theories of health (related to self-efficacy) would play a moderating role in information framework and health-related behaviors. According to the IMB model, information is the initial condition for taking healthy behaviors, we put forward the hypothesis:

*H3*: The lay theories of health moderate the effect of information frame on the interaction about mindfulness information.

## 3. Study 1

### 3.1. Participants and procedure

A total of 282 undergraduate students (24.8% male and 75.2% female) recruited from a Zhejiang university participated in the experiment in exchange for a small gift. According to the Student IDs, participants were randomly assigned to gain frame or loss frame group. More specifically, students with odd numbers at the end of their student IDs were assigned to the gain frame group, while those with even numbers were assigned to the loss frame group. Their ages ranged from 18 to 22. They all used the social media app WeChat and the features of WeChat moments and groups.

Participants were told that they would take part in a series of short, unrelated studies. The first part included the priming manipulations of information framework. The second part was introduced as a survey, which aimed to seek the willingness to interact mindfulness information. Specifically, participants were asked to view either gain framed or loss framed mindfulness information. After reading each information, they completed a self-administered questionnaire, which included dependent variables and demographic questions. The whole process takes about 12 min.

### 3.2. Research design and variables

One single-factor experimental design was used to test the hypotheses. The independent variable is the frame(gain-or loss-framed information), and the dependent variable is the willingness to interact with mindfulness information. Participants were randomly assigned to gain frame or loss frame group, and the experiment was executed online.

In order to manipulate the types of information frames, two versions of message to promote mindfulness practice were designed. Specifically, the gain-framed information highlighted the ideas about achieving the positive results (e.g., mindfulness can improve happiness; mindfulness can increase the awareness; mindfulness can help with emotions, etc.). The loss-framed information emphasized the ideas about avoiding the negative outcomes (e.g., Without mindfulness, individuals might suffer from depression or anxiety; Without mindfulness, our attention is easily to be distracted; Without mindfulness, one might suffer from negative emotions, etc.). Except for the gain or loss-framed manipulations, all other visual elements in the interface of Wechat moments, such as profile picture, screen name, and layout, remain the same.

The dependent variable is willingness to interact mindfulness information, which was measured by three items. Participants were asked to rate their agreement on a 1–7 scale with the following statements (1 = strongly disagree, 7 = strongly agree): “I’ll give this message a compliment,” “I am likely to share it with WeChat group,” and “I intend to comment on this information.” The three items are averaged to form an index for willingness to interact with mindfulness information (α = 0.797).

### 3.3. Results

#### 3.3.1. Manipulation checks

As a check of information framework manipulation, a seven-point scale (from 1 = strongly disagree to 7 = strongly agree) was used with two items: (1) The above information emphasizing the benefits of mindfulness; (2) The above information emphasizing the disadvantages of mindlessness. As expected, the score about the benefits of mindfulness in the gain-framed information condition was greater than the score in the loss-framed condition (*t* = −20.415, *p* = 0.000; *M*_*ga*in_ = 5.40, *SD_gain_* = 1.936; *M_loss_* = 1.59, *SD_loss_* = 1.152). However, the score about disadvantages of mindlessness in the loss-framed information condition was greater than the score in the gain-framed condition (*t* = 15.392, *p* = 0.000; *M*_*ga*in_ = 3.10, *SD_gain_* = 2.084; *M_loss_* = 6.08, *SD_loss_* = 1.067). Thus, the information framing manipulation was successful.

#### 3.3.2. Hypothesis testing

To test the hypotheses, the independent T-test was performed. As expected, the results show that individuals were more willing to interact with mindfulness information when the information was gain-framed (*M*_loss_ = 3.061 vs. *M*_gain_ = 3.401, *t* = −2.129, *p* = 0.034). Thus, H1 was strongly supported.

#### 3.3.3. Discussion and introduction to study 2

In Study 1, it was found that when mindfulness information is represented by gain framework, people show more willingness of interaction compared to loss framework. In a sense, mindfulness is essentially a type of preventive health behavior. Our study demonstrated the gain framework can better promote individuals participation in preventive-related health behaviors, which was consistent with the findings from Rothman and Salovey (1997). Previous studies also have shown the relative persuasiveness of the gain or loss framework depends on the characteristics of the receiver, such as perceived susceptibility (Nan et al., 2016) and independent (interdependent) self-construal (Zhang C. et al., 2021; Zhang N. et al., 2021). Therefore, on the basis of study 1, our following objective was to further explore which characteristics of the receiver influence the relative persuasiveness of the mindfulness information of gains or losses. Specifically, we test the mediating effect of health risk perception (hypothesis 2) and the moderating effect of the lay theories of health (hypothesis 3) in study 2.

## 4. Study 2

### 4.1. Design and participants

A total of 308 undergraduate students (25.3% male and 74.7% female) recruited from a university in Zhejiang Province participated in the experiment in exchange for a small gift. Their ages ranged from 18 to 22. They all used the social media app WeChat and the features of WeChat moments and groups. The participants were randomly assigned to one of two different conditions that were obtained by varying frame (gain vs. loss). The dependent variable was willingness to interact with mindfulness information as in Study 1.

### 4.2. Measures and procedure

Health risk perception. The scale of health risk perception is comprised of two dimensions, perception of susceptibility and perception of severity (Witte, 1994; Zhou and Lin, 2020). The first six items describe the perception of susceptibility (e.g., “I feel that my health is vulnerable at present”), and the last 4 items describe the perception of severity (e.g., “Illness will have a bad influence on my social life”). The participants were asked to answer the items on a 7-point Likert scale (1 = strong agreement, 7 = strong disagreement). The average score was calculated as the level of health risk perception. The higher score represent a higher level of health risk perception (α = 0.838).

The lay theories of health. The scale of the lay theories of health was developed by Bunda and Busseri (2019) with six items. Three of these items measure entity theory of health, e.g., “My health is a part of me that I cannot change very much.” The remaining three items measure incremental theory of health, e.g., “I can change even my basic level of health considerably.” The participants were asked to answer the items on a 7-point Likert scale (1 = strong agreement, 7 = strong disagreement). After reverse-scoring of the entity item ratings, the average score was calculated as the level of the incremental theory of health. The higher score represent a stronger incremental theory of health (α = 0.732).

Procedures were roughly the same as those of Study 1 except adding two variables (health risks perception and the lay theories of health). More specifically, participants were told that they would participate in a series of short, unrelated studies. The first part is a survey, which aimed to find out the lay theories of health of participants; and the next part included the priming manipulations of information framework. The third part was a survey that sought participants’ perception of health risks and their willingness to interact with mindfulness information, also including demographic questions. The whole process took about 18 min.

### 4.3. Results

#### 4.3.1. Predictors of willingness to interact with mindfulness information

Regression analyses revealed that information framing, health risk perception, and incremental theory of health were significant predictors of people’s willingness to interact with mindfulness information except entity theory of health (gender as a control variable). These results suggested that gain-framing information, and a higher level of health risk perception and incremental theory of health was more likely to promote willingness to interact with mindfulness information. However, individuals with higher entity theory of health were impossible to interact with mindfulness information (see Table 1).

**Table 1:** Predictors of willingness to interact with mindfulness information.

**Variables**	**B**	**SE**	**β**	***t***	***p***	**95% CI**
Constant	1.081	0.677		1.597	0.111	[−0.251, 2.412]
Information frame	0.372	0.152	0.138	2.445	0.015	[0.073, 0.672]
HRP	0.212	0.077	0.156	2.735	0.007	[0.059, 0.364]
Entity	0.108	0.072	0.092	1.508	0.133	[−0.033, 0.250]
Incremental	0.145	0.067	0.130	2.167	0.031	[0.013, 0.276]

The code of loss-framed information = 1; the code of gain-framed information = 2.

Abbreviation: HRP=Health Risk Perception.

#### 4.3.2. Mediated analyses of health risk perception

The results of mediator analysis with selected gender as a control variable, information framing as the independent variable, willingness to interact with mindfulness information as the dependent variable, and health risk perception as the mediator are presented in Table 2. Model 4 of the PROCESS 3.3 macro program was used to analyse the mediating effect of the above variables (Hayes, 2018). The direct effect of information framing on willingness to interact with mindfulness information was significant, β = 0.370, 95% CI: [0.069, 0.671] (confidence interval did not include 0), *p* = 0.016.The indirect effect of information framing on willingness to interact with mindfulness information through health risk perception was significant, B = −0.058, SE = 0.037, 95% CI: [−0.146, −0.003] (confidence interval did not include 0; See Table 2). Therefore, health risk perception partially mediated the relationship between information framing and willingness to interact with mindfulness information.

**Table 2:** Regression analysis of mediated model of health risk perception.

**Predictor variables**	**Model 1 (**HRP**)**				**Model 2** (Willingness of interaction)			
	***B***	***t***	***SE***	***p***	***B***	***t***	***SE***	***p***
Information framing	−0.256^*^	−2.264	0.113	0.024	0.370^*^	2.416	0.153	0.016
HRP					0.229^**^	2.963	0.077	0.003
***R***^**2**^	0.018				0.045			
***F***	2.759				4.790			
***p***	0.065				0.003			

Abbreviation: HRP=Health Risk Perception. *p < 0.05; **p < 0.01.

Interestingly, the findings show that the information frame (the code of loss-framed information is 1; the code of gain-framed information is 2) has a positive direct effect on the willingness to interact with information, that is, the gain framework is more likely to promote the willingness to interact with mindfulness information; However, in the indirect path in which information frames affect the willingness to interact with mindfulness information by health risk perception, the loss of frame information makes individuals feel higher risk and more willing to interact with mindfulness information.

#### 4.3.3. The moderated meditation analysis of the lay theories of health

**Figure 1:**
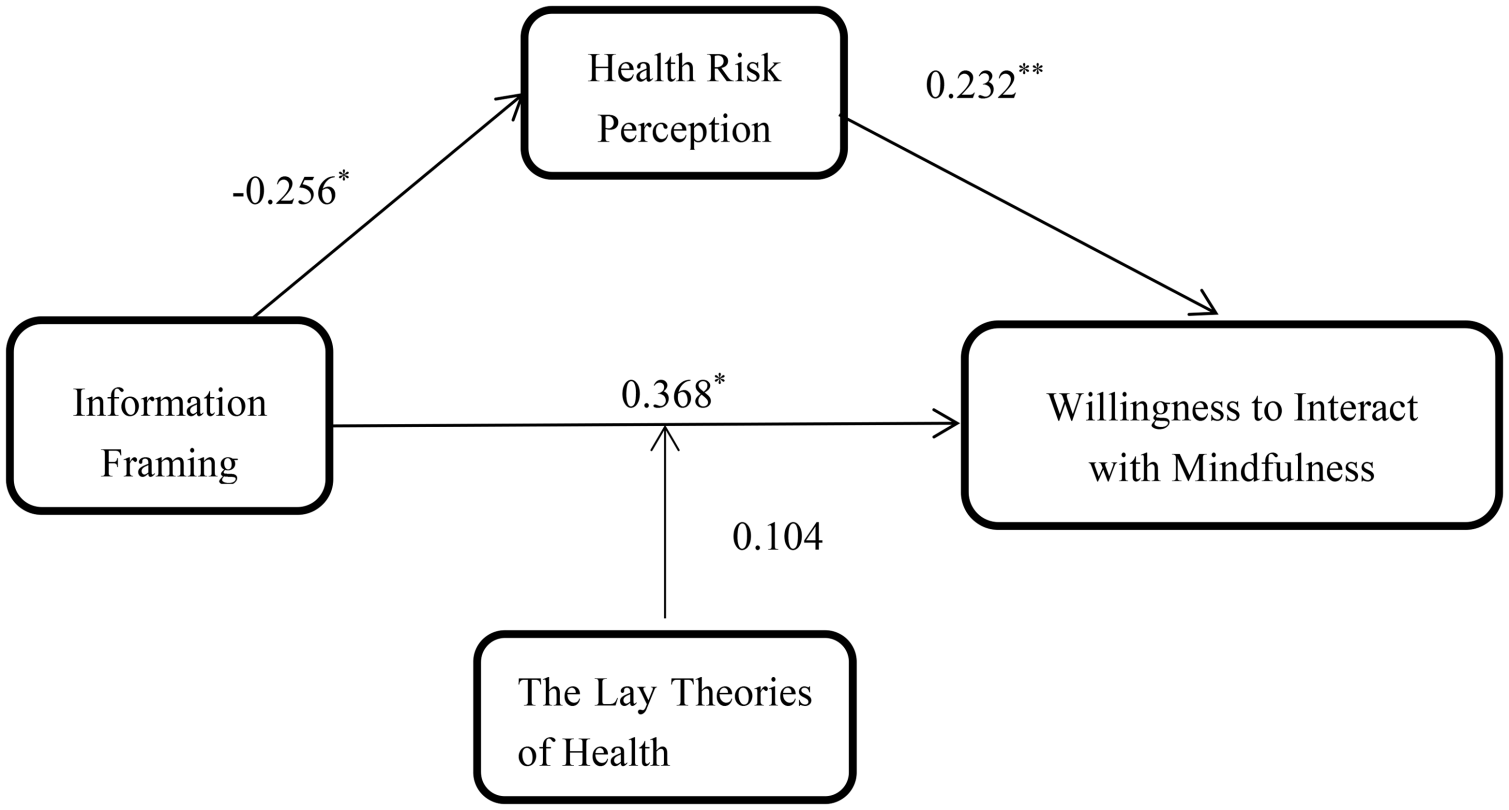
The model of a moderating effect of the lay theories of health and a mediating effect of health risk perception. The code of loss-framed information = 1; the code of gain-framed information = 2; *p < 0.05; **p < 0.01.

We employed the PROCESS proposed by Hayes (2018) to test the moderated mediation effect of the lay theories of health on the indirect effect of information framing on willingness to interact with mindfulness information *via* health risk perception. More specifically, we specified a moderated mediation model that estimates the indirect effect of X (information framing) on Y (willingness to interact with mindfulness information) *via* M (health risk perception) at different levels of V (the lay theories of health). Model 5 of the PROCESS was performed(gender was used as a control variable). The results indicated that the conditional direct effect of information framing on willingness to interact with mindfulness information was significant. When the lay theories of health is low (M − 1SD), β = 0.268, 95% CI: [−0.158, 0.693] (confidence interval include 0), *p* = 0.217; however, When the lay theories of health is high (M + 1SD), β = 0.469, 95% CI: [0.039, 0.899] (confidence interval did not include 0), *p* = 0.033. In addition, the results indicate that the indirect effects of information framing on willingness to interact mindfulness information *via* health risk perception was significant (Effect: −0.059, SE = 0.039, 95% CI: [−0.153, −0.004] (as shown in Table 3). Therefore, our findings revealed that there was a moderating effect of the lay theories of health and a mediating effect of health risk perception in the relationship between information framing and willingness to interact with mindfulness information. The model were shown in Figure 1.

**Table 3:** Moderated Mediation analysis: effects of information framing and the lay theories of health on willingness to interact with mindfulness information *via* health risk perception.

**Dependent variable**	**Conditional direct effects**				**Indirect effect**		
	Moderator	Coefficient	SE	95% CI	Coefficient	SE	95% CI
Willingness to interact information	M-SD	0.268	0.216	−0.158, 0.693	−0.059	0.039	−0.153, −0.004
	M	0.368	0.153	0.066, 0.670			
	M + SD	0.469	0.219	0.039, 0.899			

**Figure 2:**
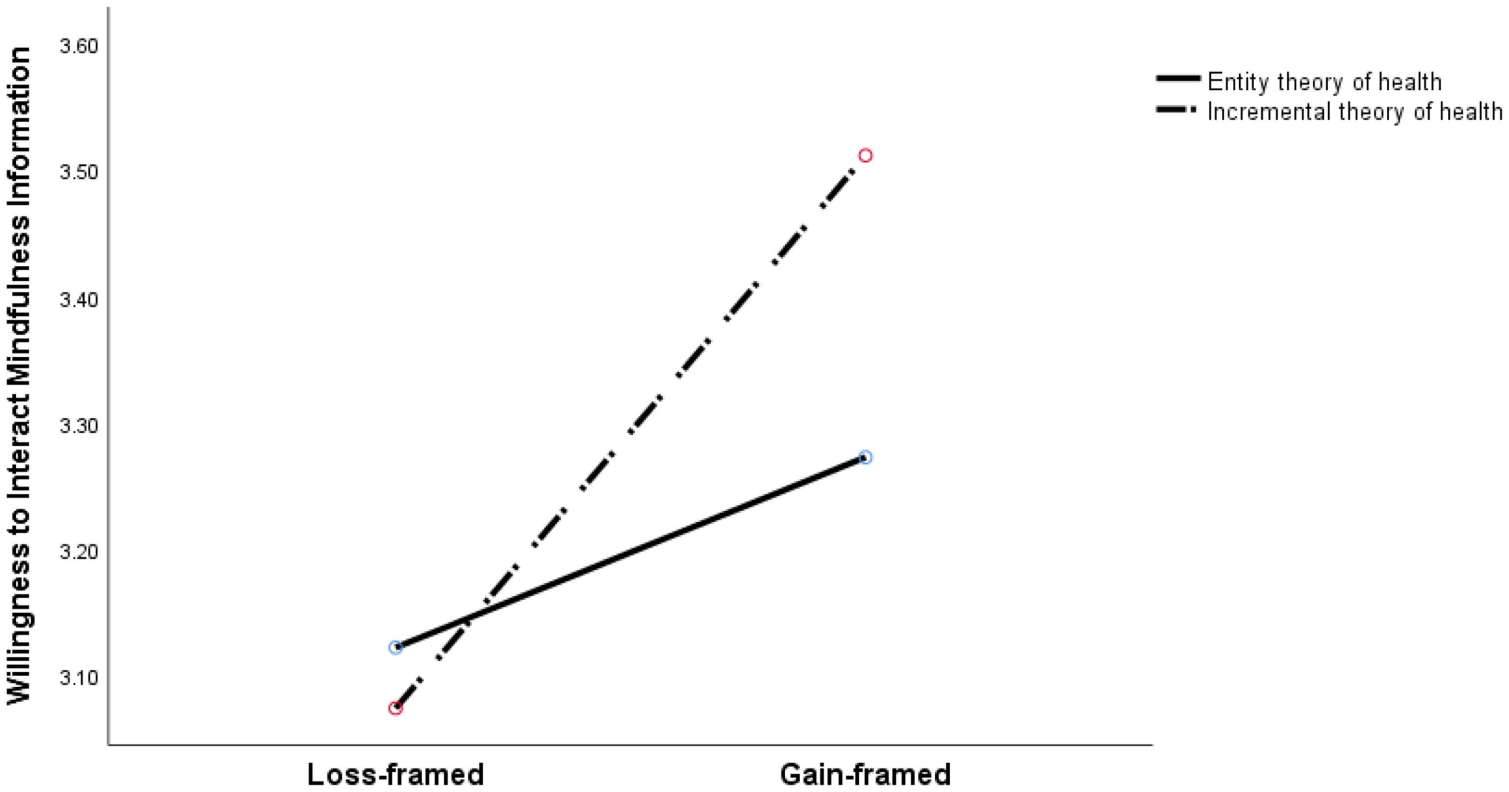
The interaction effect of information framing and the lay theories of health on willingness to interact with mindfulness information.

We then plotted the nature of the interaction of information framing and the lay theories of health which was divided into high and low groups according to the median(As shown in the Figure 2). A simple effect analysis further showed that under a low group of the lay theories of health(the entity theory), there was no significant difference in the willingness to interact with mindfulness information between the gain-framed and loss-framed information [*F*(1,302) = 0.465, *p* = 0.496]. However, under a high group of the lay theories of health(the incremental theory), the willingness to interact with mindfulness information [F(1,302) = 4.164, *p* = 0.042] was significantly more positively under the gain-framed information condition (M = 3.512, SD = 1.271) compared with the loss-framed information condition (M = 3.074, SD = 1.498).

### 4.4. Discussion

The current research demonstrated that information framing predicted individuals’ willingness to interact with mindfulness information through health risk perception as a mediating variable, and moderated by the lay theories of health. This study contributes to the research on framing theory by demonstrating that information frame could predict people’s willingness to interact with mindfulness information. Although previous research found the effect of information framing on health behaviors, it is unclear whether it could predict the willingness to interact with mindfulness information to promote healthy behaviors in others. The current study filled this gap by providing evidence that gain-framing information, a higher level of health risk perception and the incremental theory of health were important predictors of willingness to interact with mindfulness information. This research has the following main findings.

The results of two experiments show that the information framework can directly or indirectly affect the willingness to interact with mindfulness information. In experiment 1, it is found that under the condition of gain framework, people are more willing to interact with mindfulness information than under the condition of loss framework. In experiment 2, we not only verified that the information framework has a significant direct effect, but also found that the information framework can have a negatively significant indirect impact on the willingness to interact with mindfulness information through health risk perception. Loss framing which emphasizes the loss caused by not adopting a certain healthy behavior can induce fear. Some scholars have found that fear makes people more likely to perceive health risks, and fear indirectly affects attitudes toward recommended health behaviors and intentions to implement health behaviors through health risk perceptions (Nan, 2017).

The data results of experiment 2 showed that the loss framing rather than gain framing was easier to stimulate people’s risk perception which was, in turn, more likely to interact with mindfulness information with others. This is consistent with previous studies that when individuals perceive a higher level of risk, they are more sensitive to information and tend to share relevant information with others (Turner et al., 2010; Zhang et al., 2020). Risk perception is an intermediary variable, which affects people’s willingness to share information (Li and Zhang, 2021). Therefore, in the design of mindfulness information, if the audience perception risk is not started or very low, the gain-framed information is more effective; while when the audience risk perception is activated or very high, the information of loss framework is more effective. As can be seen from the above results, which effect is better, the gain framing or the loss framing? This is not only related to the “prevention-detection” behavior (Rothman and Salovey, 1997), but also to the specific situation of the individual, such as the level of risk perception. Thus it can be seen that different information frameworks induce different risk perceptions and different persuasion effects. In fact, the relationship between information frame and perceived risk is relatively complicated. Previous studies have found that when information focuses on long-term health risks, loss-framed information is more convincing, while in information that focused on short-term health risks, gain-framed information is more convincing than loss-framed information (Keyworth et al., 2018).Subsequent related research needs to be further refined on this basis.

Additionally, our results show that the lay theories of health plays an important role in message-framing effects. Specifically, the lay theories of health plays a moderating role in the direct effect of information framework on willingness to interact mindfulness information. However, this process varied depending on the level of the lay theories of health. In other words, information stressing gains may be more effective than messages stressing losses in the direct path of framing effect, but only in individuals with incremental theory. Such findings are consistent with previous research, individuals with more incremental theories of health reported stronger intentions to pursue more health-promoting behaviors than those with entity theorists. Thus, people with a higher level of incremental theory of health believe that they can improve their health status through their own efforts, and have stronger intentions to engage in healthy lifestyles such as exercising and healthy eating (Bunda and Busseri, 2019). These findings suggest that individuals’ the lay theories of health (changeable or fixed) can affect the health information interaction. In particular, individuals who think that their health can be changed to a large extent believe that their health will improve greatly over time through a mindful lifestyle. These results have great implications for health communication or the effectiveness of public health education activities. For example, if such information encourage individuals to believe that mindfulness practice can increase wellbeing based on individual efforts, such propaganda may lead them to adopt a stronger incremental lay theory, and enhance motivation to implement health-related behavior.

## 5. Implications and prospect of research

There is increasing interest in mindfulness for its potential benefits in digital media to promote human capabilities such as creativity (Chen et al., 2021, 2022a), empathy (Zhu et al., 2017a,b; Chen et al., 2021), self-identity (Thieme et al., 2013), altruism (Liu et al., 2022c) and communication skills (Chen et al., 2021, 2022b). These studies put more focus on human-centered information design. In this paper, we are pioneering to introduce information frame and the lay theories of health into predicting individual willingness to interact with mindfulness information through mobile social media. These results broaden our understanding on the underlying mechanism of the role of information frame on health behavior and information dissemination. Given that information frame, health risk perception, and the lay theories of health could be manipulated to change. Our results have important implications for designing personalized mindfulness information and developing effective health information interaction. Some strategies such as designing a gain framework for information, initiating or promoting a incremental health theory and raising the level of health risk perception could inspire other researchers to plan their own effective health information interaction. As a result, it has a significant impact on hoisting people’s health information literacy and promoting positive health outcomes.

## Data availability statement

The raw data supporting the conclusions of this article will be made available by the authors, without undue reservation.

## Ethics statement

Ethical review and approval was not required for the study on human participants in accordance with the local legislation and institutional requirements. The participants provided their written informed consent to participate in this study.

## Author contributions

XW: conceptualization, project administration, writing-original draft preparation, and methodology. BZ: investigation, project administration, and writing and editing. QG: funding acquisition and resources. WW and RZ: investigation, data curation, and software. All authors contributed to the article and approved the submitted version.

## Funding

This research was funded by the National Social Science Foundation of China (grant number: 20BXW041) and “Journalism and Communication” top 100 academic disciplines cultivation construction project of the School of Media Studies and Humanities, Zhejiang University City College.

## Conflict of interest

The authors declare that the research was conducted in the absence of any commercial or financial relationships that could be construed as a potential conflict of interest.

## Publisher’s note

All claims expressed in this article are solely those of the authors and do not necessarily represent those of their affiliated organizations, or those of the publisher, the editors and the reviewers. Any product that may be evaluated in this article, or claim that may be made by its manufacturer, is not guaranteed or endorsed by the publisher.
