# Erratum: Designing mindfulness information for interaction in social media: The role of information framing, health risk perception and lay theories of health

**DOI:** 10.3389/fpsyg.2023.1156391

**Published:** 2023-02-10

**Authors:** Frontiers Production Office

**Affiliations:** Frontiers Media SA, Lausanne, Switzerland

**Keywords:** mindfulness information, information framing, health risk perception, lay theories of health, health information

Due to a production error, the DOI for this article was incorrectly registered as 10.3389/fpsyg.2023.1041016. The correct DOI for the article is 10.3389/fpsyg.2022.1041016.

The publisher apologizes for this mistake. The original version of this article has been updated.

